# Inpatient Outcomes of Gastrointestinal Bleeding in Advanced CKD and Kidney Transplant Recipients: A National Analysis 2016-2019

**DOI:** 10.34067/KID.0000000662

**Published:** 2024-11-22

**Authors:** Mingyue He, Shaan Desai, Yichen Wang, Chien-Wen Yang, Frank Friedenberg, Avrum Gillespie

**Affiliations:** 1 Department of Internal Medicine, Temple University Hospital, Philadelphia, Pennsylvania; 2 Department of Medicine, Perelman School of Medicine, University of Pennsylvania, Philadelphia, Pennsylvania; 3 Department of Nephrology, Ochsner Medical Center, New Orleans, Los Angeles; 4 Department of Nephrology, Ochsner Clinical School, The University of Queensland, Brisbane, Queensland, Australia; 5 Section of Gastroenterology, Lewis Katz School of Medicine at Temple University, Philadelphia, Pennsylvania; 6 Section of Nephrology, Hypertension and Kidney Transplantation, Lewis Katz School of Medicine at Temple University, Philadelphia, Pennsylvania

**Keywords:** CKD, clinical epidemiology, clinical nephrology, ESKD, kidney transplantation, mortality

## Abstract

**Key Points:**

- Advanced CKD and ESKD are independent risk factors for gastrointestinal bleeding hospitalizations, angiodysplasia bleeding, and in-hospital mortality.
- Patients with ESKD with gastrointestinal bleeding exhibit significantly higher rates of adverse outcomes compared with those without CKD.
- Patients with advanced CKD and ESKD had lower rates of early endoscopy and higher rates of delayed endoscopy, with delayed endoscopy linked to increased mortality.

**Background:**

Patients with kidney disease are at increased risk for gastrointestinal bleeding (GIB). This study aimed to investigate the incidence, causes, interventions, and inpatient outcomes of GIB in patients with advanced CKD (ACKD), ESKD, and kidney transplant (KT) recipients, compared with those without CKD (NCKD).

**Methods:**

This retrospective study used the Nationwide Inpatient Sample database to identify adult patients admitted nonelectively with GIB from 2016 to 2019. Patients were stratified into five groups: ACKD (CKD stages 4 or 5), ESKD, KT, NCKD, and others (including CKD stages 1–3 and unspecified CKD). We compared outcomes across these groups and conducted subgroup analyses within the ACKD and ESKD groups to explore the association between mortality and the timing of endoscopic evaluation. Multivariate logistic regression (for binary outcomes) and linear regression (for continuous outcomes) models were used to analyze the dependent variables.

**Results:**

A total of 2,163,929 patients were included. The incidence of GIB hospitalizations was higher in the ACKD (3.2%) and ESKD (3.4%) groups and lower in the KT group (2.1%) compared with the NCKD group (2.2%). All-cause in-hospital mortality was increased in ACKD, ESKD, and KT (3.0%, 3.1%, and 2.0%, respectively) compared with NCKD (1.7%). ESKD patients had higher rates of mechanical ventilation, vasopressor support, and blood transfusion, along with prolonged and costly hospitalizations (*P* < 0.001). ACKD and ESKD groups had lower rates of early endoscopy (<24 hours) and higher rates of delayed endoscopy (>48 hours), with delayed endoscopy linked to increased mortality. ACKD and ESKD were independent risk factors for angiodysplasia bleeding, while KT was a risk factor for diverticular and esophageal bleeding.

**Conclusions:**

ACKD and ESKD are independent risk factors for GIB hospitalizations and in-hospital mortality, with delayed endoscopy further worsening outcomes. Tailored treatment plans are essential to improve outcomes in this complex population.

## Visual Abstract

**Figure:**
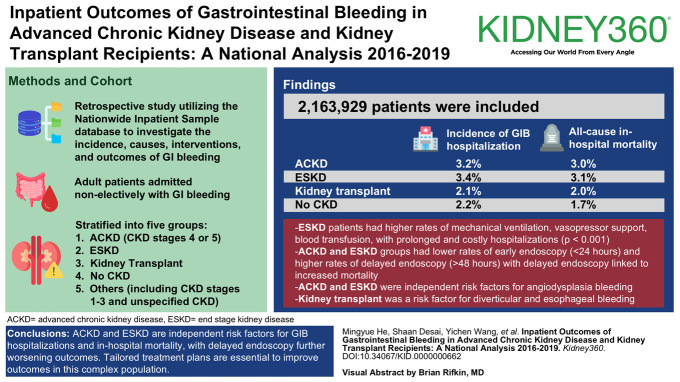

## Introduction

Gastrointestinal bleeding (GIB) is a critical medical emergency, significantly contributing to hospital admissions, mortality, and health care costs. In the United States, GIB accounts for approximately 250,000 to 530,000 hospitalizations and 15,000 to 30,000 deaths annually, with costs exceeding $3.7 billion, imposing a substantial burden on the health care system.^1–4^ There is increasing concern about the rising incidence of GIB and the associated heightened mortality and morbidity among patients with CKD,^5–7^ particularly because GIB is responsible for 3%–7% of deaths in patients with ESKD.^8–10^ Despite these concerns, the incidence of GIB hospitalizations and inpatient outcomes in CKD and ESKD populations remain unclear. In addition, the risk of GIB and related adverse outcomes in kidney transplant (KT) recipients requires further investigation.

While studies have shown mortality benefits from early endoscopy, and guidelines recommend esophagogastroduodenoscopy (EGD) within 24 hours for acute nonvariceal upper GIB,^11–14^ a national analysis found that patients with ESKD are less likely to undergo endoscopic evaluation compared to those without kidney disease.^3^ The reasons for these lower rates of endoscopy and their potential effect on the higher mortality observed in CKD and ESKD patients are not well understood, nor is the optimal timing for such evaluations in these populations.

Given the global burden of CKD, affecting approximately 700 million people worldwide,^15^ understanding the effect of GIB in this patient population is crucial. To date, no systematic study has compared the incidence of GIB hospitalizations, etiology, interventions, and outcomes among patients with advanced CKD (ACKD), ESKD, KT, and those without CKD (NCKD). This study aims to address this gap by using data from the largest publicly available inpatient database in the United States, providing health care practitioners with comprehensive insights into how ACKD, ESKD, and KT influence the clinical course and outcomes of GIB.

## Methods

### Data Source

This retrospective, observational study used the Nationwide Inpatient Sample (NIS) database, part of the health care cost and utilization project supported by the Agency for Healthcare Research and Quality.^16^ The NIS is the largest publicly available all-payer inpatient database in the United States, representing approximately a 20% stratified sample of nonfederal acute care inpatient hospitalizations nationwide. Data from 2016 to 2019 were used for this analysis.

### Ethic Statement

Institutional Review Board approval was not required because of the study's retrospective design and use of deidentified data. The research adhered to the NIS-health care cost and utilization project data-use agreements.

### Study Population

We identified all patients hospitalized with a principal diagnosis of GIB, including upper, lower, and unspecified GIB. Patients younger than 18 years and those admitted electively were excluded. The remaining patients comprised our study cohort, which was used to analyze all outcomes except the incidence of GIB hospitalizations. These patients were categorized into five groups: ACKD (CKD stages 4 or 5), ESKD, KT recipients, NCKD (patients without a diagnosis of CKD), and Others (including CKD stages 1–3 and unspecified CKD stages). International Classification of Diseases, Tenth Revision, Clinical Modification codes were used for data extraction (see Supplemental Table 1). Owing to the high proportion of unspecified CKD cases, the others group was highly heterogeneous and therefore excluded from subsequent comparative statistical analyses in this article.

To calculate the incidence of GIB hospitalizations, we identified all patients aged 18 years or older who were nonelectively hospitalized and categorized them into the five groups mentioned above.

### Study Outcomes and Variables

The primary outcome was all-cause in-hospital mortality. Secondary outcomes include (*1*) incidence of GIB hospitalizations; (*2*) etiology of GIB; (*3*) in-hospital morbidity (blood transfusion, mechanical ventilation, and vasopressor use); (*4*) endoscopy evaluations (EGD and colonoscopy); (*5*) rates of arterial embolization and surgical intervention; and (*6*) resource utilization (length of stay [LOS], total hospitalization charges). Outcomes were compared across groups. Subgroup analyses in patients with ACKD and ESKD assessed the effect of endoscopy timing on mortality.

Potential confounders included demographics (age, sex, race), socioeconomic status (median income by zip code, primary payer), hospital characteristics (bed capacity, teaching status, location), and comorbidities (*e.g*., diabetes, hypertension, obesity, congestive heart failure, coronary artery disease, chronic pulmonary disease, chronic liver disease, valvular disease, cardiac arrhythmia, atrial fibrillation, coagulopathy, abnormal international normalized ratio (INR), thrombocytopenia, nonmetastatic solid tumors, metastatic cancer). Comorbidity burden was assessed using the Charlson comorbidity index (CCI), but CCI was excluded from multivariable models because of multicollinearity concerns (variance inflation factor [VIF] >5). Data on anticoagulant and antiplatelet use, alcohol, and drug use were also considered. Detailed diagnostic codes are provided in Supplemental Table 1.

### Statistical Analysis

Statistical analyses were conducted using Stata 18.0 (StataCorp LLC, TX). The NIS’s complex sampling design required treating data as survey data, with adjustments using Agency for Healthcare Research and Quality's discharge-level weights. Proportions were compared using the chi-square test and means using ANOVA. Logistic regression was used for binary outcomes and linear regression for continuous outcomes. Multivariable regression models were constructed to calculate adjusted odds ratios (aORs) and adjusted mean differences (aMDs). Multicollinearity was assessed using the VIF, with a mean VIF of approximately 1.6, confirming no significant multicollinearity. Final multivariable models included confounders with a univariable analysis *P* value <0.2. A two-sided *P* value <0.05 was considered statistically significant.

## Results

### Patient and Hospital Characteristics

**Figure 1:**
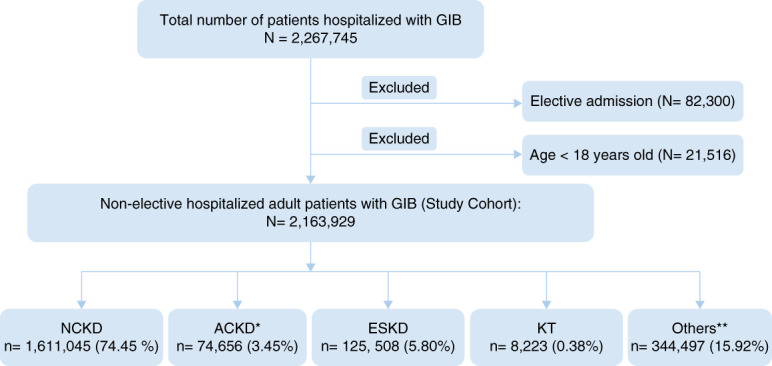
**Flow diagram of study selection.** Patients were identified from the NIS database 2016-2019. *ACKD: patients with a diagnosis of CKD stage 4 or 5. **Others include patients with a diagnosis of CKD stage 1, CKD stage 2, CKD stage 3, and unspecified CKD stages. ACKD, advanced CKD; GIB, gastrointestinal bleeding; KT, kidney transplant; NCKD, Non-CKD; NIS, Nationwide Inpatient Sample.

Figure 1 illustrates the flow diagram detailing the study’s inclusion process. The study cohort included 2,163,929 adult patients nonelectively admitted for GIB between 2016 and 2019. Of these, 74,656 (3.45%) had ACKD, 125,508 (5.8%) had ESKD, 8223 (0.38%) were KT recipients, and 1,611,045 (74.45%) had NCKD.

Table 1 summarizes the patient-level and hospital-level characteristics within the cohort. The average age was 68 years, with 47.9% female and 68.6% White. Significant differences were observed across the groups (*P* < 0.001). The ACKD group was the oldest (mean age 76 years) and had the highest proportion of patients with a CCI ≥3 (94.4%). The ESKD group had the highest proportion of Black patients (37.6%) and lower median income, with 93.5% of patients having a CCI ≥ 3. The KT group, representing the youngest demographic, had a mean age of 62 years and the lowest proportion of female patients (39.5%).

**Table 1:** Baseline characteristics of adult patients nonelectively hospitalized for gastrointestinal bleeding

Baseline Characteristics	Total (*n*=2,163,929)	NCKD (*n*=1,611,045)	ACKD (*n*=74,656)	ESKD (*n*=125,508)	KT (*n*=8223)
Age, yr, mean (95% CI)	68.1 (68.04 to 68.20)	66.1 (66.0 to 66.2)	76.2 (76.0 to 76.4)	66.7 (66.5 to 66.8)	61.6 (60.9 to 62.2)
Age, yr, median (IQR)	71 (23)	68 (24)	78 (15)	68 (17)	63 (18)
Female, *n* (%)	1,036,522 (47.9)	783,773 (48.7)	36,529 (48.9)	56,730 (45.2)	3249 (39.5)
**Race, *n* (%)**					
Asian or Pacific Islander	62,970 (2.9)	44,787 (2.8)	2635 (3.5)	5974 (4.8)	392 (4.8)
Black	348,393 (16.1)	226,996 (14.1)	14,498 (19.4)	47,216 (37.6)	2286 (27.8)
Hispanic	200,596 (9.3)	154,016 (9.6)	5308 (7.1)	17,471 (13.9)	1018 (12.4)
Native American	15,797 (0.7)	12,566 (0.8)	381 (0.5)	1280 (1.0)	56 (0.7)
Others	51,285 (2.4)	40,276 (2.5)	1545 (2.1)	3113 (2.5)	265 (3.2)
White	1,484,888 (68.6)	1,132,565 (70.3)	50,288 (67.4)	50,429 (40.2)	4206 (51.2)
**Median household income for patient's zip code, *n* (%)**					
$1–$ 49,999	660,431 (30.5)	484,763 (30.1)	22,979 (30.8)	49,036 (39.1)	2397 (29.2)
$50,000–$64,999	565,867 (26.2)	423,060 (26.3)	18,992 (25.4)	31,189 (24.9)	1934 (23.5)
$65,000–$85,999	516,313 (23.9)	385,845 (24.0)	17,940 (24.0)	26,131 (20.8)	2188 (26.6)
$86,000 or more	421,317 (19.5)	317,376 (19.7)	14,745 (19.8)	19,153 (15.3)	1705 (20.7)
**Insurance status, *n* (%)**					
Medicare	1,457,839 (67.4)	991,437 (61.5)	63,532 (85.1)	104,536 (83.3)	6273 (76.3)
Medicaid	242,793 (11.2)	211,047 (13.1)	3748 (5.0)	10,656 (8.5)	395 (4.8)
Private, including HMO	378,255 (17.5)	328,814 (20.4)	6644 (8.9)	9300 (7.4)	1505 (18.3)
Self-pay	85,259 (3.9)	79,908 (5.0)	732 (1.0)	1029 (0.8)	50 (0.6)
**Hospital bed size, *n* (%)**					
Small	435,599 (20.1)	329,942 (20.5)	14,573 (19.5)	21,437 (17.1)	1087 (13.2)
Medium	664,543 (30.7)	498,296 (30.9)	22,598 (30.3)	36,824 (29.3)	2238 (27.2)
Large	1,063,787 (49.2)	782,968 (48.6)	37,485 (50.2)	67,247 (53.6)	4898 (59.6)
**Hospital location/teaching status, *n* (%)**					
Rural	179,390 (8.3)	138,389 (8.6)	6376 (8.5)	5849 (4.7)	396 (4.8)
Urban nonteaching	517,612 (23.9)	392,612 (24.4)	17,051 (22.8)	25,880 (20.6)	1433 (17.4)
Urban teaching	1,466,927 (67.8)	1,080,045 (67.0)	51,229 (68.6)	93,767 (74.7)	6394 (77.8)
**Hospital region, *n* (%)^a^**					
Northeast	409,199 (18.9)	306,904 (19.1)	14,939 (20.0)	22,077 (17.6)	1749 (21.3)
Midwest	475,199 (22.0)	338,319 (21.0)	18,112 (24.3)	26,507 (21.1)	2066 (25.1)
South	858,647 (39.7)	647,318 (40.2)	28,929 (38.8)	52,487 (41.8)	2975 (36.2)
West	420,884 (19.5)	318,342 (19.8)	12,677 (17.0)	24,449 (19.5)	1433 (17.4)
**CCI, *n* (%)**					
0	339,737 (15.7)	340,253 (21.1)	0 (0.0)	0 (0.0)	0 (0.0)
1	476,281 (22.0)	477,030 (29.6)	0 (0.0)	0 (0.0)	0 (0.0)
2	368,301 (17.0)	328,009 (20.4)	4196 (5.6)	8145 (6.5)	1300 (15.8)
≥3	979,611 (45.3)	465,753 (28.9)	70,460 (94.4)	117,363 (93.5)	6923 (84.2)
Hypertension, *n* (%)	1,538,337 (71.1)	1,032,519 (64.1)	69,139 (92.6)	118,555 (94.5)	7156 (87.0)
Diabetes, *n* (%)	689,861 (31.9)	408,239 (25.3)	41,501 (55.6)	76,485 (60.9)	4146 (50.4)
Obesity, *n* (%)	279,796 (12.9)	195,742 (12.2)	11,661 (15.6)	16,630 (13.3)	900 (10.9)
Chronic heart failure, *n* (%)	496,622 (23.0)	247,940 (15.4)	40,434 (54.2)	57,119 (45.5)	2307 (28.1)
Cardiac arrhythmia, *n* (%)	675,795 (31.2)	434,016 (26.9)	34,387 (46.1)	43,501 (34.7)	2678 (32.6)
Atrial fibrillation, *n* (%)	517,828 (23.9)	314,637 (19.5)	29,168 (39.1)	35,757 (28.5)	2204 (26.8)
Coronary artery disease, *n* (%)	641,605 (29.7)	388,745 (24.1)	35,648 (47.8)	56,115 (44.7)	3000 (36.5)
Valvular disease, *n* (%)	213,363 (9.9)	124,695 (7.7)	13,266 (17.8)	16,881 (13.5)	998 (12.1)
Chronic pulmonary disease, *n* (%)	496,189 (22.9)	343,797 (21.3)	20,433 (27.4)	31,490 (25.1)	1077 (13.1)
Chronic liver disease, *n* (%)	313,770 (14.5)	249,551 (15.5)	7428 (10.0)	19,454 (15.5)	826 (10.0)
Metastasis cancer, *n* (%)	57,777 (2.7)	46,076 (2.9)	1486 (2.0)	1519 (1.2)	138 (1.7)
Solid tumor without metastasis, *n* (%)	113,606 (5.3)	88,124 (5.5)	3270 (4.4)	4016 (3.2)	237 (2.9)
Alcohol use disorder, *n* (%)	269,626 (12.5)	241,818 (15.0)	2949 (4.0)	5673 (4.5)	133 (1.6)
Substance use disorder, *n* (%)	89,370 (4.1)	77,491 (4.8)	1090 (1.5)	3765 (3.0)	197 (2.4)
Abnormal INR, *n* (%)	56,046 (2.6)	35,282 (2.2)	3150 (4.2)	3577 (2.9)	252 (3.1)
Coagulopathy, *n* (%)	283,691 (13.1)	200,897 (12.5)	11,490 (15.4)	21,663 (17.3)	1161 (14.1)
Thrombocytopenia, *n* (%)	176,360 (8.2)	126,306 (7.8)	6921 (9.3)	14,772 (11.8)	603 (7.3)
On antiplatelet agent, *n* (%)	127,888 (5.9)	87,158 (5.4)	4957 (6.6)	8284 (6.6)	465 (5.7)
On anticoagulant agent, *n* (%)	352,720 (16.3)	231,185 (14.4)	16,268 (21.8)	17,935 (14.3)	1611 (19.6)

ACKD, advanced CKD; CCI, Charlson comorbidity index; CI, confidence interval; INR, international normalized ratio; IQR, interquartile range; KT, kidney transplant; NCKD, non-CKD.

a Regions of hospital in Nationwide Inpatient Sample: refer to Supplemental Table 2.

**Figure 2:**
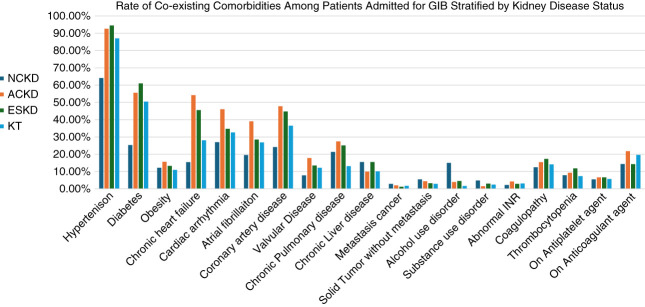
**Prevalence of chronic comorbidities among patients admitted for GIB with different kidney disease status.** INR, international normalized ratio.

Figure 2 compares the prevalence of chronic comorbidities among the groups. Patients with ACKD and ESKD were more likely than those without CKD to have hypertension, diabetes, obesity, chronic heart failure, cardiac arrhythmia, atrial fibrillation, coronary artery disease, valvular disease, chronic pulmonary disease, and coagulopathy.

### Outcomes

#### All-Cause In-Hospital Mortality and Trends

The overall all-cause in-hospital mortality rate was 1.9%, with 1.7% in NCKD, 3.0% in ACKD, 3.1% in ESKD, and 2.0% in KT (*P* < 0.001; Table 2). Adjusted mortality odds ratios were 1.33 for ACKD (95% confidence interval [CI], 1.19 to 1.48; *P* < 0.001), 1.94 for ESKD (95% CI, 1.78 to 2.13; *P* < 0.001), and 1.73 for KT (95% CI, 1.22 to 2.46; *P* = 0.002; Table 3). Mortality trends remained stable across all groups over the study period (*P* trend > 0.05).

**Table 2:** Inpatient outcomes and interventions for gastrointestinal bleeding across different groups

Inpatient Outcomes and Interventions	Total (*n*=2,163,929)	Non-CKD (*n*=1,611,045)	ACKD (*n*=74,656)		ESKD (*n*=125,508)	KT (*n*=8223)	*P* Value
All cause in hospital mortality, *n* (%)	41,764 (1.9)	27,549 (1.7)	2232 (3.0)		3866 (3.1)	163 (2.0)	<0.001
Mechanical ventilation, *n* (%)	46,741 (2.2)	33,832 (2.1)	1620 (2.2)		4104 (3.3)	232 (2.8)	<0.001
Vasopressor, *n* (%)	12,551 (0.6)	8216 (0.5)	448 (0.6)		1494 (1.2)	83.8746 (1.0)	<0.001
Blood transfusion, *n* (%)	708,038 (32.7)	491,691 (30.5)	32,423 (43.4)		52,249 (41.6)	2931 (35.6)	<0.001
Any endoscopy, *n* (%)	1,576,206 (72.8)	1,169,941 (72.6)	54,566 (73.1)		92,236 (73.5)	6177 (75.1)	<0.001
Mean interval to any endoscopic eval, d	1.5	1.4	1.8		1.9	1.6	<0.001
Mean interval to EGD eval, d	1.5	1.4	1.8		1.9	1.6	<0.001
Mean interval to colonoscopy eval, d	2.2	2.1	2.6		2.7	2.1	<0.001
**The interval between admission to any endoscopic eval**							
Early endoscopic evaluation <24 h, *n* (%)	710,396 (45.1)	544,023 (46.5)	21,701 (39.8)	35,575 (38.6)		2728 (44.2)	<0.001
Endoscopic evaluation between 24 and 48 h, *n* (%)	238,480 (15.1)	171,513 (14.7)	8872 (16.3)	15,477 (16.8)		1080 (17.5)	<0.001
Endoscopic evaluation >48 h, *n* (%)	627,488 (39.8)	454,288 (38.8)	23,993 (44.0)	41,183 (44.7)		2368 (38.3)	<0.001
Arterial embolism	37,003 (1.7)	27,227 (1.7)	963 (1.3)	2786 (2.2)		192 (2.3)	<0.001
Surgical resection	18,393 (0.9)	14,822 (0.9)	493 (0.7)	904 (0.7)		69 (0.8)	<0.001
Mean LOS, d	4.3	4.0	5.4	5.7		4.7	<0.001
Mean total hospitalization charges, USD	48,334	45,768	54,961	70,169		57,833	<0.001

ACKD, advanced CKD; EGD, esophagogastroduodenoscopy; KT, kidney transplant; LOS, length of stay.

**Table 3:** aOR for inpatient outcomes of gastrointestinal bleeding among patients with kidney disease compared with those without CKD

Inpatient Outcomes	ACKD	ESKD	KT
**In-hospital mortality**			
aOR (95% CI)	1.33^a^ (1.19 to 1.48^a^)	1.94^a^ (1.78 to 2.13^a^)	1.73^a^ (1.22 to 2.46^a^)
*P* value	<0.001^a^	<0.001^a^	0.002^a^
**Mechanical ventilation**			
aOR (95% CI)	0.98 (0.86 to 1.10)	1.42^a^ (1.30 to 1.54^a^)	1.47^a^ (1.09 to 1.98^a^)
*P* value	0.7	<0.001^a^	0.01^a^
**Vasopressor**			
aOR (95% CI)	0.71^a^ (0.56 to 0.89^a^)	1.36^a^ (1.17 to 1.57^a^)	1.25 (0.76 to 2.05)
*P* value	0.003^a^	<0.001^a^	0.38
**Transfusion**			
aOR (95% CI)	1.24^a^ (1.19 to 1.29^a^)	1.27^a^ (1.23 to 1.32^a^)	1.07 (0.96 to 1.19)
*P* value	<0.001^a^	<0.001^a^	0.22
**LOS**			
aMD (95% CI)	0.75^a^ (0.67 to 0.83^a^)	1.12^a^ (1.02 to 1.21^a^)	0.48^a^ (0.25 to 0.71^a^)
*P* value	<0.001^a^	<0.001^a^	<0.001^a^
**Total hospitalization charges**			
aMD (95% CI)	3605^a^ (2474 to 4737^a^)	16,661^a^ (15,371 to 17,950^a^)	8732^a^ (5280 to 12,185^a^)
*P* value	<0.001^a^	<0.001^a^	<0.001^a^

ACKD, advanced CKD; aMD, adjusted mean difference; aOR, adjusted odds ratios; CI, confidence interval; KT, kidney transplant; LOS, length of stay.

a Values represent statistical significance.

The multivariable regression model identified several independent risk factors associated with increased odds of all-cause in-hospital mortality, including age (adjusted odds ratios [aOR], 1.04; 95% CI, 1.04 to 1.04; *P* < 0.001), chronic heart failure (aOR, 1.75; 95% CI, 1.65 to 1.86; *P* < 0.001), cardiac arrhythmia (aOR, 2.28; 95% CI, 2.12 to 2.45; *P* < 0.001), chronic pulmonary disease (aOR, 1.10; 95% CI, 1.04 to 1.16; *P* < 0.001), chronic liver disease (aOR, 2.34; 95% CI, 2.20 to 2.49; *P* < 0.001), coagulopathy (aOR, 3.50; 95% CI, 3.27 to 3.75; *P* < 0.001), abnormal INR (aOR, 1.43; 95% CI, 1.26 to 1.63; *P* < 0.001), solid tumor (aOR, 1.88; 95% CI, 1.72 to 2.06; *P* < 0.001), and metastatic cancer (aOR, 2.57; 95% CI, 2.30 to 2.87; *P* < 0.001) (see Supplemental Table 3).

#### Incidence of GIB Hospitalizations and Trends

**Figure 3:**
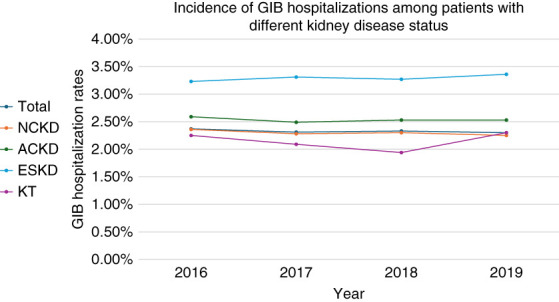
**Incidence of GIB hospitalizations across groups over years (2016–2019; adjusted by age and sex).** A significant decrease in GIB hospitalizations was observed from 2016 to 2019 in the NCKD group (*P* trend < 0.001), but not in the ACKD, ESKD, or KT groups (*P* trend > 0.05).

From 2016 to 2019, a total of 93 million patients older than 18 years were nonelectively hospitalized, including 75 million NCKD, 2,374,350 ACKD, 3,746,000 ESKD, and 402,280 KT patients. Among these, 2,163,929 were admitted with a principal diagnosis of GIB, resulting in an overall incidence of GIB hospitalizations of 2.3%. By group, the rates were 2.2% in NCKD, 3.2% in ACKD, 3.4% in ESKD, and 2.1% in KT. After adjusting for covariates, the aORs for GIB hospitalizations were higher in ACKD (aOR, 1.12; 95% CI, 1.10 to 1.14; *P* < 0.001) and ESKD (aOR, 1.37; 95% CI, 1.35 to 1.39; *P* < 0.001), but lower in KT (aOR, 0.88; 95% CI, 0.83 to 0.92; *P* < 0.001). A significant decrease in GIB hospitalizations was observed from 2016 to 2019 in the NCKD group (*P* trend < 0.001), but not in the ACKD, ESKD, or KT groups (*P* trend > 0.05, see Figure 3).

For the incidence of GIB hospitalizations, the multivariable regression model identified several independent risk factors, including age (aOR, 1.02; 95% CI, 1.02 to 1.02; *P* < 0.001), race/ethnicity (*e.g*., Black: aOR, 1.23; 95% CI, 1.21 to 1.24, compared with White; *P* < 0.001), coagulopathy (aOR, 1.98; 95% CI, 1.94 to 2.01; *P* < 0.001), use of antiplatelet agents (aOR, 1.65; 95% CI, 1.62 to 1.67; *P* < 0.001), use of anticoagulants (aOR, 1.50; 95% CI, 1.49 to 1.52; *P* < 0.001), abnormal INR (aOR, 1.94; 95% CI, 1.90 to 1.98; *P* < 0.001), chronic liver disease (aOR, 2.20; 95% CI, 2.18 to 2.23; *P* < 0.001), alcohol use (aOR, 1.73; 95% CI, 1.71 to 1.75; *P* < 0.001), and other comorbidities (see Supplemental Table 4).

#### Etiology of GIB

Among all patients, nonvariceal upper GIB (UGIB) was the most common cause (38.7%), followed by unspecified GIB (37.0%, defined as the presence of melena, hematemesis, or unspecified GIB without an identified bleeding source), lower GIB (LGIB, 22.9%), and variceal UGIB (1.4%). Notably, 27% of patients did not undergo endoscopic evaluation to determine the source of GIB, potentially contributing to the high percentage of unspecified GIB cases. Excluding unspecified GIB, the most common causes in NCKD and KT were gastric/duodenal, diverticular, and esophageal bleeding, while in ACKD and ESKD, they were gastric/duodenal, angiodysplasia, and diverticular bleeding (see Supplemental Table 5).

Multivariable regression showed higher odds of nonvariceal UGIB in ACKD (aOR, 1.09; 95% CI, 1.05 to 1.13; *P* < 0.001) and ESKD (aOR, 1.14; 95% CI, 1.11 to 1.17; *P* < 0.001) compared with NCKD. Angiodysplasia bleeding was more common in ACKD (aOR, 1.57; 95% CI, 1.48 to 1.66; *P* < 0.001) and ESKD (aOR, 1.93; 95% CI, 1.84 to 2.03; *P* < 0.001), while KT patients had higher odds of diverticular (aOR, 1.56; 95% CI, 1.37 to 1.78; *P* < 0.001) and esophageal bleeding (aOR, 1.43; 95% CI, 1.19 to 1.71; *P* < 0.001) compared with NCKD (see Supplemental Table 6).

#### Morbidity

Blood transfusions were required in 32.7% of patients, with higher rates in the ACKD (aOR, 1.24; 95% CI, 1.19 to 1.29; *P* < 0.001) and ESKD groups (aOR, 1.27; 95% CI, 1.23 to 1.32; *P* < 0.001) compared with NCKD. Mechanical ventilation was required in 2.2% of patients, with higher odds in ESKD (aOR, 1.42; 95% CI, 1.30 to 1.54; *P* < 0.001) and KT (aOR, 1.47; 95% CI, 1.09 to 1.98; *P* = 0.01) compared with NCKD. Vasopressor support was needed in 0.6% of cases, with increased usage among patients with ESKD (aOR, 1.36; 95% CI, 1.17 to 1.57; *P* < 0.001) compared with NCKD. No significant differences were observed between KT and NCKD in transfusions or vasopressor use (Table 3).

#### Interventions

##### Endoscopic Evaluation

Overall, 73% of patients underwent endoscopic evaluation, including diagnostic and therapeutic EGD and/or colonoscopy procedures (Table 2). Patients with ESKD were less likely to receive endoscopy (aOR, 0.97; 95% CI, 0.93 to 0.99; *P* = 0.04). Early endoscopy (within 24 hours of admission) was less common in ESKD (aOR, 0.83; 95% CI, 0.80 to 0.85; *P* < 0.001 for early EGD; aOR, 0.67; 95% CI, 0.63 to 0.71; *P* < 0.001 for early colonoscopy), while late endoscopy (after 48 hours) was more frequent (aOR, 1.2; 95% CI, 1.16 to 1.23; *P* < 0.001 for late EGD; aOR, 1.44; 95% CI, 1.39 to 1.50; *P* < 0.001 for late colonoscopy). Similar trends were seen in ACKD. KT patients showed no significant differences in endoscopy rates compared with NCKD, except for a higher likelihood of early colonoscopy (Table 4).

**Table 4:** aOR for GIB interventions among patients with kidney disease compared with those without CKD

GIB Interventions	ACKD			ESKD			KT		
	aOR	95% CI	*P* Value	aOR	95% CI	*P* Value	aOR	95% CI	*P* Value
**Any Endoscopy**	1.01	0.97 to 1.05	0.76	0.97^a^	0.93 to 0.99^a^	0.04^a^	1	0.89 to 1.12	0.997
Any EGD	1.001	0.97 to 1.05	0.66	0.97^a^	0.94 to 1.00	0.051	1.01	0.90 to 1.13	0.92
Any colonoscopy	0.88^a^	0.84 to 0.91^a^	<0.001^a^	0.79^a^	0.76 to 0.82^a^	<0.001^a^	1.08	0.97 to 1.20	0.17
**Early endoscopy(<24 h)**	0.91^a^	0.87 to 0.94^a^	<0.001^a^	0.83^a^	0.80 to 0.85^a^	<0.001^a^	0.94	0.85 to 1.04	0.26
Early EGD (<24 h)	0.91^a^	0.88 to 0.94^a^	<0.001^a^	0.83^a^	0.80 to 0.85^a^	<0.001^a^	0.95	0.85 to 1.05	0.278
Early colonoscopy (<24 h)	0.74^a^	0.69 to 0.79^a^	<0.001^a^	0.67^a^	0.63 to 0.71^a^	<0.001^a^	1.23^a^	1.06 to 1.43^a^	0.006^a^
**Late endoscopy (>48 h)**	1.11^a^	1.07 to 1.15^a^	<0.001^a^	1.2^a^	1.16 to 1.23^a^	<0.001^a^	1	0.91 to 1.11	0.97
Late EGD (>48 h)	1.11^a^	1.07 to 1.15^a^	<0.001^a^	1.2^a^	1.16 to 1.23^a^	<0.001^a^	1	0.91 to 1.11	0.97
Late colonoscopy (>48 h)	1.3^a^	1.24 to 1.36^a^	<0.001^a^	1.44^a^	1.39 to 1.50^a^	<0.001^a^	0.9	0.79 to 1.01	0.08
Arterial embolization	0.6^a^	0.51 to 0.70^a^	<0.001^a^	1.06	0.96 to 1.17	0.244	1.18	0.86 to 1.63	0.311
Surgical intervention	0.72^a^	0.58 to 0.89^a^	0.003^a^	0.71^a^	0.60 to 0.84^a^	<0.001^a^	0.74	0.43 to 1.28	0.28

ACKD, advanced CKD; aOR, adjusted odds ratios; CI, confidence interval; EGD, esophagogastroduodenoscopy; GIB, gastrointestinal bleeding; KT, kidney transplant.

a Values represent statistical significance.

In the study cohort, among all patients with GIB, multivariate regression analysis demonstrated that endoscopy was associated with lower mortality (aOR, 0.35; 95% CI, 0.33 to 0.37; *P* < 0.001). Moreover, early endoscopy was linked to reduced mortality, while late endoscopy was associated with increased mortality (see Supplemental Table 3). To account for potential confounding due to hemodynamic instability at admission, which might delay endoscopy and increase mortality, we conducted a repeated analysis excluding patients who required intubation or vasopressor support within 24 hours of admission. The repeated multivariate analysis produced results consistent with the primary findings, further validating the initial conclusions (*P* < 0.001 for all; Supplemental Table 7).

##### Subgroup Analysis on the Basis of Timing of Endoscopy in ESKD and ACKD

To determine whether the timing of endoscopic evaluation influences mortality rates among patients with ESKD, a focused subgroup analysis was conducted. In patients with ESKD, mortality was 2.55% for endoscopy within 24 hours, 1.29% for 24–48 hours, and 4.17% for >48 hours (see Table 5). Multivariate regression analysis revealed that any endoscopic evaluation, particularly early endoscopy (within 24 hours), was associated with lower mortality (aOR, 0.38; 95% CI, 0.33 to 0.44; *P* < 0.001 for any endoscopy; aOR, 0.77; 95% CI, 0.66 to 0.91; *P* = 0.002 for early EGD; aOR, 0.65; 95% CI, 0.47 to 0.92; *P* = 0.014 for early colonoscopy), while late endoscopy (after 48 hours) was linked to higher mortality (aOR, 1.86; 95% CI, 1.60 to 2.17; *P* < 0.001 for late EGD; aOR, 2.22; 95% CI, 1.66 to 2.96; *P* < 0.001 for late colonoscopy; Table 6). Similar results were seen in ACKD. Excluding patients requiring early intubation or vasopressors within 24 hours of admission yielded results consistent with the primary analysis, thereby validating the findings (Table 6).

**Table 5:** Mortality rates by timing of endoscopy for each group

Timing of Endoscopy, h	NCKD, *n *(%)	ACKD, *n* (%)	ESKD, *n* (%)	KT, *n* (%)
<24	6419 (1.18)	497 (2.29)	907 (2.55)	41 (1.50)
24–48	1201 (0.70)	102 (1.15)	200 (1.29)	4 (0.34)
>48	12,357 (2.72)	1034 (4.31)	1717 (4.17)	78 (3.30)

ACKD, advanced CKD; KT, kidney transplant; NCKD, non-CKD.

**Table 6:** Relationship between mortality and timing of endoscopy: subgroup multivariable regression analysis in ESKD and advanced CKD group

Endoscopy Evaluation	Before Excluding Sick Patients*			After Excluding Sick Patients		
	aOR	95% CI	*P* Value	aOR	95% CI	*P* Value
**Subgroup analysis in ESKD**						
Any endoscopy	0.38^a^	0.33 to 0.44^a^	<0.001^a^	0.39^a^	0.33 to 0.46^a^	<0.001^a^
*Any EGD*	0.38^a^	0.33 to 0.44^a^	<0.001^a^	0.39^a^	0.33 to 0.45^a^	<0.001^a^
*Any colonoscopy*	0.41^a^	0.33 to 0.52^a^	<0.001^a^	0.44^a^	0.35 to 0.56^a^	<0.001^a^
Early endoscopy, <24 h	0.77^a^	0.66 to 0.91^a^	0.001^a^	0.72^a^	0.61 to 0.85^a^	<0.001^a^
*Early EGD*	0.77^a^	0.66 to 0.91^a^	0.002^a^	0.72^a^	0.61 to 0.86^a^	<0.001^a^
*Early colonoscopy*	0.65^a^	0.47 to 0.92^a^	0.014^a^	0.67^a^	0.47 to 0.96^a^	0.028^a^
Late endoscopy, >48 h	1.87^a^	1.60 to 2.17^a^	<0.001^a^	1.95^a^	1.65 to 2.29^a^	<0.001^a^
*Late EGD*	1.86^a^	1.60 to 2.17^a^	<0.001^a^	1.94^a^	1.65 to 2.29^a^	<0.001^a^
*Late colonoscopy*	2.22^a^	1.66 to 2.96^a^	<0.001^a^	2.13^a^	1.58 to 2.88^a^	<0.001^a^
**Subgroup analysis in ACKD**						
Any endoscopy	0.35^a^	0.29 to 0.43^a^	<0.001^a^	0.36^a^	029 to 0.44^a^	<0.001^a^
*Any EGD*	0.35^a^	0.29 to 0.43^a^	<0.001^a^	0.36^a^	029 to 0.44^a^	<0.001^a^
*Any colonoscopy*	0.4^a^	0.30 to 0.54^a^	<0.001^a^	0.43^a^	0.32 to 0.58^a^	<0.001^a^
Early endoscopy, <24 h	0.7^a^	0.57 to 0.87^a^	0.001^a^	0.68^a^	0.55 to 0.85^a^	0.001^a^
*Early EGD*	0.7^a^	0.57 to 0.87^a^	0.001^a^	0.68	0.55 to 0.85^a^	0.001^a^
*Early colonoscopy*	0.74	0.49 to 1.13	0.162	0.80	0.53 to 1.22	0.307
Late endoscopy, >48 h	2.08^a^	1.70 to 2.55^a^	<0.001^a^	2.11^a^	1.71 to 2.61^a^	<0.001^a^
*Late EGD*	2.08^a^	1.70 to 2.55^a^	<0.001^a^	2.11^a^	1.70 to 2.61^a^	<0.001^a^
*Late colonoscopy*	1.97^a^	1.40 to 2.78^a^	<0.001^a^	1.81^a^	1.28 to 2.56^a^	0.001^a^

* Patients who required intubation or vasopressor support within 24 hours of admission. ACKD, advanced CKD; aOR, adjusted odds ratios; CI, confidence interval; EGD, esophagogastroduodenoscopy.

a Values represent statistical significance.

##### Arterial Embolization and Surgical intervention

A total of 1.7% of patients required arterial embolization, and 0.9% needed surgical intervention. Patients with ACKD had a reduced likelihood of requiring these interventions compared with NCKD (aOR, 0.6; 95% CI, 0.51 to 0.70; *P* < 0.001 for embolization; aOR, 0.72; 95% CI, 0.58 to 0.89; *P* = 0.003 for surgery). ESKD also showed reduced odds for surgical intervention (aOR, 0.71; 95% CI, 0.60 to 0.84; *P* < 0.001). No significant differences were noted between KT and NCKD (Table 3).

#### Resource Utilization

Patients with ACKD, ESKD, or KT had significantly longer hospital stays and higher hospitalization charges compared with those without CKD. The mean LOS for all GIB patients was 4.3 days, with adjusted analyses showing extended stays for ACKD, ESKD, and KT patients (adjusted mean difference [aMD], 0.75 days; 95% CI, 0.67 to 0.83 for ACKD; aMD, 1.12 days; 95% CI, 1.02 to 1.21 for ESKD; aMD, 0.48 days; 95% CI, 0.25 to 0.71 for KT). Similarly, the mean total hospitalization charge was $48,334, with higher costs for ACKD, ESKD, and KT patients (aMD, $3,605; 95% CI, $2474 to $4737 for ACKD; aMD, $16,661; 95% CI, $15,731 to $17,950 for ESKD; aMD, $8,732; 95% CI, $5280 to $12,185 for KT; Table 3).

## Discussion

This study, using the largest inpatient database in the United States, provides a comprehensive analysis of GIB in more than two million adult patients hospitalized from 2016 to 2019. By comparing patients with ACKD, ESKD, and KT with those without CKD, our study offers the most exhaustive comparative analysis in this area to date.

Our study revealed four significant findings. First, ACKD and ESKD are independent risk factors for GIB, nonvariceal UGIB, angiodysplasia bleeding, and in-hospital mortality. Second, patients with ESKD and GIB exhibited significantly worse outcomes, including higher rates of mechanical ventilation, pressor support, blood transfusions, and longer, more costly hospitalizations compared with those without CKD. Third, both patients with ACKD and ESKD had lower rates of early endoscopy (<24 hours) and higher rates of late endoscopy (>48 hours), with both delayed and decreased early endoscopy identified as independent risk factors for mortality. Finally, while KT patients had a lower incidence of GIB hospitalizations, they experienced higher in-hospital mortality and had comparable intervention rates with NCKD patients. KT was also found to be an independent risk factor for diverticular and esophageal bleeding. These insights underscore the significant effect of kidney disease and transplantation on the clinical outcomes of GIB.

Our study revealed that patients with ACKD and ESKD had significantly higher incidences of GIB hospitalizations, with aORs of 1.12 and 1.37, respectively, compared with NCKD patients. While these findings are in line with previous research^17–20^ reporting increased incidences of UGIB with CKD progression^17^ and higher rates of ulcer bleeding among patients with CKD and ESKD,^18,19^ we acknowledge that direct comparisons should be made with caution due to differences in study populations and definitions. The elevated risk of GIB in CKD patients may be attributed to several factors, including gastric mucosal damage from the uremic environment, uremic platelet dysfunction and coagulopathy, systemic and local chronic circulatory dysfunction, and elevated serum gastrin levels.^21–23^ In addition, the use of antiplatelet agents and anticoagulants has been proposed as a potential contributor to the increased GIB risk in this population. In our cohort, patients with ACKD and ESKD had a higher prevalence of coronary artery disease and atrial fibrillation, conditions that often require anticoagulant and antiplatelet therapy. We also observed a higher use of antiplatelet agents in these groups compared with NCKD. Importantly, our analysis identified anticoagulant and antiplatelet use as independent risk factors for GIB hospitalizations. However, the exact mechanisms linking GIB risk and renal function remain unclear and warrant further investigation.

We also found that in-hospital mortality was significantly higher in patients with ACKD and ESKD at 3.0% and 3.1%, respectively, compared with 1.7% in NCKD patients. Our analysis confirmed that both ACKD and ESKD are independent risk factors for in-hospital mortality with aORs of 1.33 and 1.94, respectively. These findings are consistent with prior studies, including a meta-analysis by Hagendorn *et al*.^24^, which included over 400,000 patients and found that ESKD increases mortality by 2.5 times and CKD by 1.8 times in GIB cases compared with those with normal renal function. Similarly, a prior NIS study by Garg *et al*.^3^ reported a 3% mortality rate among patients with ESKD. The elevated mortality in patients with ACKD and ESKD likely results from a multifactorial interplay of factors. These patients tend to be older, frailer, and burdened with multiple comorbidities, with over 90% having CCI scores above three in our baseline data. Our multivariate analysis highlights the associations between higher mortality and conditions such as older age, chronic heart failure, cardiac arrhythmia, chronic lung disease, and coagulopathy, which are more common in patients with ACKD and ESKD. In addition, the higher mortality in patients with ESKD may be attributed to the greater severity of GIB in this group. Our findings indicate that 1.73% of patients with ESKD required intubation or vasopressor support within 24 hours of admission compared with 1.24% of NCKD patients, suggesting a more severe clinical presentation of GIB in patients with ESKD. Moreover, the timing of endoscopic evaluations emerged as a critical factor in patient outcomes. Our analysis highlighted delayed endoscopy in patients with ACKD and ESKD, with significantly higher mortality observed in those undergoing endoscopy more than 48 hours after admission. Multivariate analysis showed that early endoscopy (<24 hours) was associated with reduced mortality, while delayed endoscopy (>48 hours) was linked to increased mortality. These findings are in line with international guidelines recommending early endoscopy (within 24 hours) for acute nonvariceal UGIB,^13^ although the optimal timing of endoscopy may differ for patients with CKD and ESKD compared with the general population. Factors influencing the timing of these evaluations—such as prior endoscopic assessments, severe electrolyte imbalances, pulmonary edema, patient stability, resource availability, or physician discretion—were not captured in our study, which is a limitation inherent to its retrospective design. While the timing of endoscopy should be individualized, the discrepancies observed in the real-world practice compared with recommended guidelines highlight an area for further investigation. Understanding the reasons for delayed endoscopic evaluations in patients with ESKD and ACKD is crucial for improving guidelines adherence and enhancing patient outcomes. Further research should explore the optimal timing of endoscopy and develop strategies to optimize this timing for patients with ACKD and ESKD.

Interestingly, we identified the use of anticoagulants and antiplatelets as independent risk factors for the incidence of GIB hospitalizations but not for mortality, while coagulopathy and abnormal INR were independent risk factors for both incidence and mortality. This discrepancy likely reflects the reversible nature of anticoagulants and antiplatelets, which can be withheld during active GIB, in contrast to the more complex and time-consuming reversal of coagulopathy and abnormal INR. These findings challenge the assumption that anticoagulation or antiplatelet agents invariably worsen outcomes in high-bleeding-risk populations and offer valuable insights into their risks and benefits.

Another important finding from our study is the variation in GIB causes among different patient groups. Excluding unspecified GIB, the most common causes in NCKD and KT were gastric/duodenal, diverticular, and esophageal bleeding, while in ACKD and ESKD, they were gastric/duodenal, angiodysplasia, and diverticular bleeding. Notably, the incidence of angiodysplasia bleeding decreased after kidney transplantation, suggesting a link between uremic factors and the development of this condition. Also worth noting, unspecified GIB accounted for 37% of all GIB admissions. Among these cases, 64% underwent endoscopy, 0.9% arterial embolization, and 0.3% surgery, but the bleeding source remained unidentified. These cases could represent obscure GIB, defined as recurrent or persistent GIB without an identifiable cause on EGD, colonoscopy, or radiographic imaging.^25^ Our analysis reveals a higher incidence of obscure GIB in patients with ACKD (aOR, 1.19; 95% CI, 1.12 to 1.28, *P* < 0.001) and ESKD (aOR, 1.41; 95% CI, 1.34 to 1.49, *P* < 0.001), aligning with findings from previous studies.^26^ The higher incidence of obscure GIB in patients with CKD underscores the need for targeted diagnostic strategies in this population.

In the KT population, our study observed a decreased incidence of GIB hospitalizations but increased mortality and prolonged, costly hospitalizations. This lower incidence may reflect the selective nature of the transplant population because patients at high risk of GIB may not be considered suitable candidates for transplantation. However, the higher mortality suggests that KT patients remain vulnerable, likely because of their heavy burden of comorbidities and immunosuppressed status. Moreover, KT was identified as an independent risk factor for diverticular and esophageal bleeding, highlighting the need for vigilant monitoring and management of conditions such as constipation and gastroesophageal reflux disease in this population.

While our study benefits from a large, representative sample size and comprehensive scope, several limitations must be acknowledged. The use of an administrative database limits access to specific clinical details, such as the underlying cause of kidney disease or the reasons for delayed endoscopy evaluation. In addition, the identification of clinical conditions and procedures relied on the accuracy of diagnosis and procedure codes reported by hospitals, making the database susceptible to inaccuracies or missing codes. Furthermore, the retrospective observational design also limits our ability to establish causality, despite adjustments for confounders in the analysis.

Our study highlights the significant risks associated with GIB in patients with ACKD, ESKD, and KT recipients. These populations are particularly vulnerable to adverse outcomes, including heightened mortality and prolonged, costly hospitalizations. The findings provide valuable insights for health care providers, emphasizing the importance of proactive, individualized treatment plans to improve outcomes in this medically complex patient population.

## Data Availability

All data supporting the findings of this study are included in the manuscript and/or supporting information. The data are openly available in the NIS database, 2016–2019, accessible at https://hcup-us.ahrq.gov/db/nation/nis/nisdbdocumentation.jsp.

## Supplemental Material

Supplemental Table 1. ICD‐10‐CM codes for diseases and procedures.

Supplemental Table 2. Regions of hospital in NIS.

Supplemental Table 3. Independent risk factors for in-hospital all-cause mortality.

Supplemental Table 4. Independent risk factors for incidence of GIB hospitalizations.

Supplemental Table 5. Distribution of different causes of GIB among patients with various kidney disease status.

Supplemental Table 6. Adjusted odds ratios for GIB causes in patients with ACKD, ESKD, and KT recipients compared to NCKD patients.

Supplemental Table 7. The relationship of mortality and timing of endoscopic in all hospitalized patients with GIB.

## References

[1] Abougergi MS. Epidemiology of upper gastrointestinal hemorrhage in the USA: is the bleeding slowing down? Dig Dis Sci. 2018;63(5):1091–1093. doi:10.1007/s10620-018-4951-5 29397492

[2] Charilaou P Devani K Enjamuri D Radadiya D Reddy CM Young M. Epidemiology of lower GI bleed in the United States—an update from the National inpatient survey 20052014: 559. Am J Gastroenterol. 2018;113:S319–S321. doi:10.14309/00000434-201810001-00559

[3] Garg R Parikh MP Chadalvada P, . Lower rates of endoscopy and higher mortality in end-stage renal disease patients with gastrointestinal bleeding: a propensity matched National Study. J Gastroenterol Hepatol. 2022;37(3):584–591. doi:10.1111/jgh.15771 34989024

[4] Peery AF Crockett SD Murphy CC, . Burden and cost of gastrointestinal, liver, and pancreatic diseases in the United States: update 2018. Gastroenterology. 2019;156(1):254–272.e11. doi:10.1053/j.gastro.2018.08.063 30315778 PMC6689327

[5] Lin Y Li C Waters D Kwok CS. Gastrointestinal bleeding in chronic kidney disease patients: a systematic review and meta-analysis. Ren Fail. 2023;45(2):2276908. doi:10.1080/0886022X.2023.2276908 37955109 PMC10796123

[6] Wuerth BA Rockey DC. Changing epidemiology of upper gastrointestinal hemorrhage in the last decade: a nationwide analysis. Dig Dis Sci. 2018;63(5):1286–1293. doi:10.1007/s10620-017-4882-6 29282637

[7] Geyer M Stamenic I Bühler H Bertschinger P. Epidemiology of gastrointestinal bleeding in the elderly. Praxis. 2006;95(19):757–765. doi:10.1024/0369-8394.95.19.757 16722204

[8] Kuo CC Kuo HW Lee IM Lee CT Yang CY. The risk of upper gastrointestinal bleeding in patients treated with hemodialysis: a population-based cohort study. BMC Nephrol. 2013;14(1):15. doi:10.1186/1471-2369-14-15. 23324652 PMC3558322

[9] Spinelli G Tomasello G Damiani F, . Endoscopic findings in chronic renal failure: review of literature. Acta Med Mediterr. 2012;28:261–265.

[10] Wasse H Gillen DL Ball AM, . Risk factors for upper gastrointestinal bleeding among end-stage renal disease patients. Kidney Int. 2003;64(4):1455–1461. doi:10.1046/j.1523-1755.2003.00225.x 12969166

[11] Weissman S Aziz M Bangolo AI, . Relationships of hospitalization outcomes and timing to endoscopy in non-variceal upper gastrointestinal bleeding: a nationwide analysis. World J Gastrointest Endosc. 2023;15(4):285–296. doi:10.4253/wjge.v15.i4.285 37138938 PMC10150287

[12] Garg SK Anugwom C Campbell J, . Early esophagogastroduodenoscopy is associated with better outcomes in upper gastrointestinal bleeding: a nationwide study. Endosc Int Open. 2017;5(5):E376–E386. doi:10.1055/s-0042-121665 28512647 PMC5432117

[13] Barkun AN Almadi M Kuipers EJ, . Management of nonvariceal upper gastrointestinal bleeding: guideline recommendations from the International Consensus Group. Ann Intern Med. 2019;171(11):805–822. doi:10.7326/M19-1795 31634917 PMC7233308

[14] Mullady DK Wang AY Waschke KA. AGA clinical practice update on endoscopic therapies for non-variceal upper gastrointestinal bleeding: expert review. Gastroenterology. 2020;159(3):1120–1128. doi:10.1053/j.gastro.2020.05.095 32574620

[15] GBD Chronic Kidney Disease Collaboration. Global, regional, and national burden of chronic kidney disease, 1990-2017: a systematic analysis for the Global Burden of Disease Study 2017. Lancet. 2020;395(10225):709–733. doi:10.1016/S0140-6736(20)30045-3 32061315 PMC7049905

[16] NIS Database Documentation (ahrq.gov). https://hcup-us.ahrq.gov/db/nation/nis/nisdbdocumentation.jsp

[17] Liang CC Wang SM Kuo HL, . Upper gastrointestinal bleeding in patients with CKD. Clin J Am Soc Nephrol. 2014;9(8):1354–1359. doi:10.2215/CJN.09260913 24903385 PMC4123397

[18] Luo JC Leu HB Huang KW, . Incidence of bleeding from gastroduodenal ulcers in patients with end-stage renal disease receiving hemodialysis. CMAJ. 2011;183(18):E1345–E1351. doi:10.1503/cmaj.110299 22083684 PMC3255160

[19] Tsai TJ Chen WC Huang YT, . Hemodialysis increases the risk of lower gastrointestinal bleeding and angiodysplasia bleeding: a nationwide population study. Gastroenterol Res Pract. 2020;2020:7206171. doi:10.1155/2020/7206171 32190042 PMC7072111

[20] Mahady SE Polekhina G Woods RL, . Association between CKD and major hemorrhage in older persons: data from the aspirin in reducing events in the elderly randomized trial. Kidney Int Rep. 2023;8(4):737–745. doi:10.1016/j.ekir.2023.01.012 37069989 PMC10105042

[21] Krishnan A Sigamani R Venkataraman J. Gastrointestinal evaluation in chronic kidney diseases. J Nephrol Ther. 2011;1(3):110. doi:10.4172/2161-0959.1000110

[22] Chung SY Barnes JL Astroth KS. Gastrointestinal microbiota in patients with chronic kidney disease: a systematic review. Adv Nutr. 2019;10(5):888–901. doi:10.1093/advances/nmz028 31165878 PMC6743837

[23] Kringen MK Narum S Lygren I, . Reduced platelet function and role of drugs in acute gastrointestinal bleeding. Basic Clin Pharmacol Toxicol. 2011;108(3):194–201. doi:10.1111/j.1742-7843.2010.00643.x 21118353

[24] Hágendorn R Farkas N Vincze Á, . Chronic kidney disease severely deteriorates the outcome of gastrointestinal bleeding: a meta-analysis. World J Gastroenterol. 2017;23(47):8415–8425. doi:10.3748/wjg.v23.i47.8415 29308001 PMC5743512

[25] Raju GS Gerson L Das A Lewis B, American Gastroenterological Association (AGA) Institute technical review on obscure gastrointestinal bleeding. Gastroenterology. 2007;133(5):1697–1717. doi:10.1053/j.gastro.2007.06.007 17983812

[26] Muftah M Mulki R Dhere T Keilin S Chawla S. Diagnostic and therapeutic considerations for obscure gastrointestinal bleeding in patients with chronic kidney disease. Ann Gastroenterol. 2019;32(2):113–123. doi:10.20524/aog.2018.0341 30837783 PMC6394262

